# Novel heat flux controlled surface cooling for hypersonic flight

**DOI:** 10.1038/s41598-023-40281-8

**Published:** 2023-08-11

**Authors:** Fabian Hufgard, Christian Duernhofer, Stefanos Fasoulas, Stefan Loehle

**Affiliations:** High Enthalpy Flow Diagnostics Group (HEFDiG), Institute of Space Systems, University of Stuttgart, Pfaffenwaldring 29, 70569 Stuttgart, Germany

**Keywords:** Aerospace engineering, Characterization and analytical techniques

## Abstract

This paper presents a new method in theory and experiment to adjust the transpiration cooling based on the actual measured heat flux. This is particularly useful in extreme heating environments, e.g. atmospheric entry flight or combustion chamber applications. In such environments, usually cooling is set constant based on the vehicle design, yet a mass efficient and performant cooling is sought after. We present a method with real-time surface heat flux determination of the transpiration cooled wall and an automatic adjustment of the cooling. The heat flux is determined based on a system identification process. The heat flux measurement itself is derived non-intrusively from the measurement of pressure inside the plenum, i.e. the region between mass flow controller and porous wall. The particular advantage of this system is that the heat shield material is not weakened by any sensor system and its performance is optimized with respect to cooling needed at a certain heating level. Another new feature of the pressure heat flux transformation is the attenuation of a destabilizing positive feedback loop, where the transpiration cooling controller’s output (i.e. mass flow rate) strongly influences its input (i.e. plenum pressure). We describe the identification of the model parameters for the heat flux determination, which are found and verified by a calibration approach. The controlled cooling was demonstrated in a hot air plasma flow with a reference heat flux of up to 1.4 MW/m$^2$. The results show the performance and verify the applicability in a real flight environment.

## Introduction

High heating environments require an appropriate thermal protection system. In spaceflight, the challenge is to protect the spacecraft from overheating on its return to Earth during the atmospheric entry maneuver. Typically, materials are used which consume the energy by chemical and thermal decomposition, which requires high safety margins. More general, there is currently no material available which sustains heat fluxes in high enthalpy air flows above 1 MW/m$^2$ without being significantly weakened. Thus, new concepts are currently a major field of research to advance spaceflight including return to Earth.

We study here the fundamental aspects of materials and cooling mechanisms for future space flight. The particular problem is the entry of vehicles into the Earth’s atmosphere. Here, heat flux reaches several MW/m$^2$, but it should be considered that this problem is transient. The maximum heat flux is only occurring for a short time. The total duration of a typical ballistic entry is about 20 min, but the actual critical hot phase duration is less than about 5 min. Thus, an optimization of the heat shield with respect to this transient process can have an enormous impact on the overall mass budget of a mission.

One possibility studied since the advent of porous ceramic materials is named *transpiration cooling*. This active thermal protection technique uses the idea to cool the material below its thermal limits^1–3^. Here, a gaseous or liquid coolant is fed through a porous wall material into the hot gas region. This has two effects. Firstly, the wall is actively cooled by the coolant itself^4^. Secondly, the coolant mixes into the boundary layer, and by that reduces the boundary layer temperature, leading to a lower convective surface heat flux^1,5,6^. Both effects are boosted when increasing the coolant mass flow rate^1,5,6^. However, a high mass flow rate also produces undesirable effects. A large coolant consumption requires a large coolant reservoir, thus more vehicle mass and volume. It furthermore triggers the onset of boundary layer transition earlier and shortens its extent resulting downstream in overshoots and higher heat transfer rates^7^. Ideally, the coolant mass flow rate is adjusted during flight to a level that is optimal for the actual surface heat flux. This is what we have achieved and present within this paper. The technology, which we named Cooling Adjustment for Transpiration Systems (CATS), is foreseen for flight testing during the flight experiment HIFLIER1, scheduled for June 2023^8^. The cooling adjustment is realized using the heat flux measurement based on the measurement of the plenum pressure. The characteristics and validity have been studied in detail by the authors^9–15^. This paper presents the automatic cooling adjustment based on the heat flux measurement. Essentially, the plenum pressure is a measure for surface heat flux and the coolant mass flow rate is adjusted by the CATS controller to this measured surface heat flux.

## Theoretical approach

**Figure 1:**
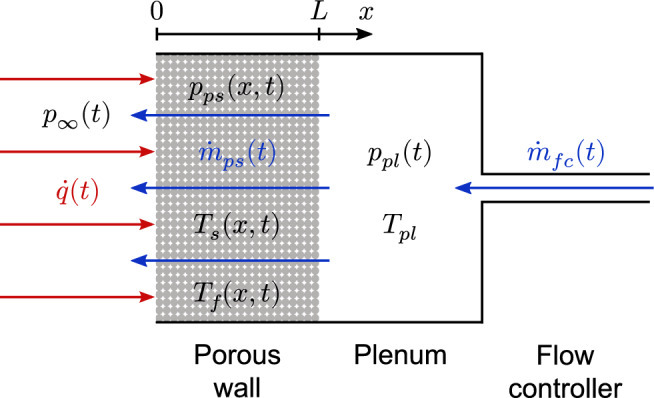
Schematic of a transpiration cooling system.

A theoretical analysis of the problem requires the coupled heat and mass transfer between the outer incoming heat flux $\dot{q}(t)$ and the measured pressure inside the plenum $p_{pl}$. Figure 1 shows the system of interest.

The pressure gradient over a porous wall of thickness *L* is given by the Darcy–Forchheimer equation^4^

1 $$\begin{aligned} p_{ps}(x,t)\frac{\partial p_{ps}(x,t)}{\partial x} = \frac{\mu _f(x,t)\,\dot{m}_{ps}(t)\,R\,T_f(x,t)}{K_D\,A} + \frac{\dot{m}_{ps}^2(t)\,R\,T_f(x,t)}{K_F\,A^2}, \qquad 0<x<L \end{aligned}$$

with the time *t*, the spatial variable *x*, the pressure inside the porous wall $p_{ps}$, the fluid viscosity $\mu _f$ and temperature $T_f$, the mass flow rate $\dot{m}_{ps}$, the specific gas constant for an ideal gas *R*, the permeability coefficients $K_D$ and $K_F$, and the porous wall cross sectional area *A*. Integration of Eq. (1) over *x* and the introduction of the plenum pressure $p_{pl}$ at $x=L$ as well as the constant ambient pressure $p_{\infty }$ at $x=0$ we obtain

2 $$\begin{aligned} \frac{p_{pl}^2(t) - p_{\infty }^2}{2} = \int _0^L\left( \frac{\mu _f(x,t)\,\dot{m}_{ps}(t)\,R\,T_f(x,t)}{K_D\,A} + \frac{\dot{m}_{ps}^2(t)\,R\,T_f(x,t)}{K_F\,A^2} \right) dx . \end{aligned}$$

If a heat flux affects the surface, the temperature of the porous wall will increase. This causes an increase in fluid temperature and viscosity inside the porous wall, which according to Eq. (2) will eventually lead to an increased plenum pressure. This means that one can determine the surface heat flux from the measurement of the plenum pressure. We have studied this approach extensively in the past^9–15^.

In the present study, we suggest to use the surface heat flux information to control the mass flow rate to adjust the cooling performance depending on the incoming heat flux value. The main difficulty is that the increase of the mass flow rate also causes a plenum pressure rise (cf. Eq. (2)). This results in a positive feedback loop on the plenum pressure, which drives the controller instability (see “Closed loop verification”). Thus, a simple follow-up controller using the plenum pressure signal as the control deviation is not useful. However, we found an approach to circumvent this difficulty so that we can use the plenum pressure in real-time to adjust the amount of cooling. Here, the variation in mass flow rate is taken into account. The resulting heat flux signal is used as the input into a simple proportional controller which adjusts the set point of the mass flow controller (MFC). This is the so-called cooling adjustment for transpiration systems (CATS) approach. In the following, we present the theoretical approach required for CATS.

The energy equations describe the heat transfer in the porous wall and are given by

3a $$\begin{aligned} (1-\Phi )\,\rho _s \,c_{p,s}\,\frac{\partial T_s(x,t)}{\partial t} = (1-\Phi )\,\lambda _s \,\frac{\partial ^2 T_s(x,t)}{\partial x^2} - h_v\,\left( T_s(x,t)-T_f(x,t)\right) , \quad 0<x<L, \end{aligned}$$

for the solid and

3b $$\begin{aligned} \Phi \, \rho _f \,c_{p,f}\,\left( \frac{\partial T_f(x,t)}{\partial t} + v(t) \frac{\partial T_f(x,t)}{\partial x}\right) = \Phi \,\lambda _f \frac{\partial ^2 T_f(x,t)}{\partial x^2} + h_v\,\left( T_s(x,t)-T_f(x,t)\right) , \quad 0<x<L, \end{aligned}$$

for the fluid^4^. We introduced the thermal conductivity $\lambda$, density $\rho$, specific heat capacity $c_p$, the volumetric heat transfer coefficient $h_v$, the Darcy velocity *v*, and the porosity $\Phi$. The subscripts ($_s$) and ($_f$) assign the variables to the solid and fluid respectively. Solving both equations for $h_v\,(T_s(x,t)-T_f(x,t))$ allows us to join Eqs. (3a) and (3b) into the following single equation,

4 $$\begin{aligned} \lambda _{s,eff}\, \frac{\partial ^2 T_s(x,t)}{\partial x^2} = \rho _{s,eff} \, c_{p,s} \,\frac{\partial T_s(x,t)}{\partial t} + \Phi \,\rho _f \,c_{p,f} \,\frac{\partial T_f(x,t)}{\partial t} + \frac{{\dot{m}}_{ps}(t)\, \Phi \,c_{p,f}}{A} \frac{\partial T_f(x,t)}{\partial x} . \end{aligned}$$

Here, we neglect conduction through the fluid and substitute the Darcy velocity with $v={\dot{m}}_{ps}/\rho _f\,A$. We also substituted the effective material properties $\rho _{s,eff} = (1-\Phi )\,\rho _s$ and $\lambda _{s,eff} = (1-\Phi )\,\lambda _s$. Since we intend to adjust the mass flow rate through the MFC ${\dot{m}}_{fc}(t)$, the mass flow rate through the porous sample ${\dot{m}}_{ps}(t)$ is also a function of time. We develop Eq. (4) by assuming local thermal equilibrium (LTE), i.e. $T_f(x,t) = T_s(x,t) = T(x,t)$, and substitution of the heat flux within the porous wall $-\dot{q}_{ps}=\lambda _{s,eff} \,\partial T / \partial x$ in the left side of the equation. Further we substitute the density of the working fluid, which is gaseous nitrogen, with the ideal gas law, i.e. $\rho _f = p / R\,T$. Real gas effects are negligible for the expected temperature and pressure ranges. With these assumptions we obtain

5 $$\begin{aligned} -\frac{\partial \dot{q}_{ps}(x,t)}{\partial x} = \left( \rho _{s,eff} \, c_{p,s} + \Phi \,\frac{p_{ps}(x,t)}{R\,T(x,t)} \,c_{p,f}\right) \,\frac{\partial T(x,t)}{\partial t} + \frac{{\dot{m}}_{ps}(t)\, \Phi \,c_{p,f}}{A} \frac{\partial T(x,t)}{\partial x}. \end{aligned}$$

Equation (5) is further simplified by assuming a lumped wall which allows the integration over *x*. This results finally in a formulation of surface heat flux dependent only on plenum and sample parameters:

6 $$\begin{aligned} \dot{q}(t) = \left( \rho _{s,eff} \, c_{p,s} + \Phi \,\frac{{\bar{p}}_{ps}(t)}{R\,{\bar{T}}(t)} \,c_{p,f}\right) \,L\,\frac{d {\bar{T}}(t)}{d t} + \frac{{\dot{m}}_{ps}(t)\, \Phi \,c_{p,f}}{A} \left( \bar{T}(t)-T_{pl}\right) \end{aligned}$$

with the lumped wall temperature ${\bar{T}}(t)$, the mean gas pressure in the porous wall ${\bar{p}}_{ps}(t)$, and the net surface heat flux that is absorbed by the wall $\dot{q}_{ps}(x=0,t)=\dot{q}(t)$. We apply a Cauchy boundary condition with the assumptions that the back wall is adiabatic, i.e. $\dot{q}_{ps}(x=L,t)=0$, and the inbound fluid temperature equals the fluid temperature in the plenum $T_{pl}$, which is constant, i.e. $T(x=L,t)=T_{pl}=const$. Some of the assumptions, e.g. the lumped system or the LTE are met better or worse depending on the chosen porous material. The used C/C material is an ideal candidate in this sense, because it features a very high volumetric heat transfer coefficient justifying the LTE assumption and also an acceptably high thermal conductivity of 13 W/(m K)^16^. Furthermore, we apply a particular calibration method which inherently considers the experimental constraints. The calibration approach is discussed below. Although these assumptions might simplify the whole system too much for a thermodynamic analysis, it turns out to be fully sufficient for the goal of this study: an accurate in situ control of the cooling through non-intrusive heat flux determination.

To realize a mass flow controller dependent on heat flux, the time dependent variables being $\bar{p}_{ps}(t)$, ${\dot{m}}_{ps}(t)$ and ${\bar{T}}(t)$ have to be determined (cf. Eq. (6)). The axial pressure distribution inside a cooled porous wall is given by^17^

7 $$\begin{aligned} p_{ps}(x) = \sqrt{\frac{p_{\infty }^2-p_{pl}^2}{L}\,x+p_{pl}^2} . \end{aligned}$$

Assuming that this distribution develops instantaneously we can find a time-dependent pressure formulation based only on the plenum and the ambient pressure. For this we integrate Eq. (7) over *x* and divide by the wall length. This yields

8 $$\begin{aligned} \bar{p}_{ps}(t) = \, \frac{2}{3}\,\frac{p_{pl}^3(t)-p_{\infty }^3(t)}{p_{pl}^2(t)-p_{\infty }^2(t)} . \end{aligned}$$

The mass flow rate through the porous wall is given by^15^

9 $$\begin{aligned} {\dot{m}}_{ps}(t)= \,{\dot{m}}_{fc}(t)-\frac{V_{pl}}{R\,T_{pl}}\frac{dp_{pl}(t)}{dt} \end{aligned}$$

with the plenum volume $V_{pl}$. The mean temperature ${\bar{T}}(t)$ can be found by solving the Darcy equation for

10 $$\begin{aligned} \mu (t)\, {\bar{T}}(t) = \frac{p_{pl}^2(t)-p_{\infty }^2(t)}{2} \, \frac{K_D\,A}{{\dot{m}}_{ps}(t)\,R\,L} . \end{aligned}$$

The product $\mu (t)\, {\bar{T}}(t)$ is a bijective function of ${\bar{T}}$ when approximating the fluid viscosity $\mu (t)$ using e.g. Sutherland’s Law. We implemented this calculation of ${\bar{T}}$ from the product $\mu (t)\, {\bar{T}}(t)$ using a simple look-up table. $K_D$ is the porous material’s permeability coefficient. We do not use the full Darcy–Forchheimer equation to solve for ${\bar{T}}$, because $K_F$ is orders of magnitude bigger than $K_D$ and the mass flux ${\dot{m}}_{ps}/A$ is quite small, which results in a negligible Forchheimer term.

Equations (8) to (10) require the input of the variables $p_{pl}(t)$, $p_{\infty }(t)$, ${\dot{m}}_{fc}(t)$, and $T_{pl}$, which are the directly measured values in the experiment. Note the feature that the determination of surface heat flux requires these four measurements as input, which are exclusively non-intrusive. No particular sensor must be mounted or attached inside the porous wall itself and the coolant flow through the porous wall is therefore not disturbed. In addition, these calculations are computationally cheap, which enables the heat flux determination in real-time.

The crucial porous material parameter is the volumetric heat transfer coefficient. It boosts the temperature response of the fluid to a solid temperature increase. According to the causal relation explained on page 2, this drives the sensitivity of plenum pressure to surface heat flux, on which the whole CATS approach hinges. This is another reason why the high volumetric heat transfer coefficient of the C/C material renders it a favorable material choice^16^.

For simplicity, we merge the constant parameters in Eq. (6) into the parameters

11a $$\begin{aligned} a&= \rho _{s,eff} \, c_{p,s}\,L \end{aligned}$$

11b $$\begin{aligned} b&= \frac{\Phi \,c_{p,f}\,L}{R} \end{aligned}$$

11c $$\begin{aligned} c&= \frac{\Phi \,c_{p,f}}{A} \end{aligned}$$

so Eq. (6) results in

12 $$\begin{aligned} \dot{q}(t) = a\,\frac{d {\bar{T}}(t)}{d t} + b\, \frac{\bar{p}_{ps}(t)}{{\bar{T}}(t)}\,\frac{d {\bar{T}}(t)}{d t} + c\,{\dot{m}}_{ps}(t) \left( {\bar{T}}(t)-T_{pl}\right) . \end{aligned}$$

Equation (12) is applied for the real-time determination of the surface heat flux. The model parameters *a*, *b*, and *c* are calculated from material properties and geometry data using Eq. (11). Due to the simplifications and assumptions made in the derivation of Eq. (12), the usage of the analytically calculated model parameters would introduce a bias. This challenge is avoided by the introduction of a system identification approach with an additional calibration step where we identify the model parameters of the actual sensor system. Another advantage of a calibration step is that the knowledge of the material parameters $\rho _{s,eff}$, $c_{p,s}$, $\Phi$, and $c_{p,f}$ is not required anymore. The calibration approach is detailed in “Calibration of heat flux determination model”.

As mentioned, the heat flux signal is used to control the transpiration cooling to reduce the thermal load. The CATS controller’s input is the heat flux determined with Eq. (12) adjusting then the set point of the mass flow rate at the MFC ${\dot{m}}_{fc,set}$. For this, we chose a P-controller, because we aim for a linear increase of cooling with heat flux. Therefore, the mass flow rate at the MFC is defined by

13 $$\begin{aligned} {\dot{m}}_{fc,set} = {\dot{m}}_{fc,0} + e\cdot \frac{{\dot{m}}_{fc,max}-{\dot{m}}_{fc,0}}{\dot{q}_{max} } \end{aligned}$$

with the control deviation $e=\dot{q}_{det}-\dot{q}_{set}$, the initial mass flow rate ${\dot{m}}_{fc,0}$, the MFC’s maximum mass flow rate ${\dot{m}}_{fc,max}$, and the maximum expected heat flux $\dot{q}_{max}$. In Eq. (13), $k_p = \left( {\dot{m}}_{fc,max}-{\dot{m}}_{fc,0}\right) /\dot{q}_{max}$ is the P-controller’s proportional gain and ${\dot{m}}_{fc,0}$ the offset. We introduced this offset, because Eq. (10) requires ${\dot{m}}_{ps}>0$.

**Figure 2:**
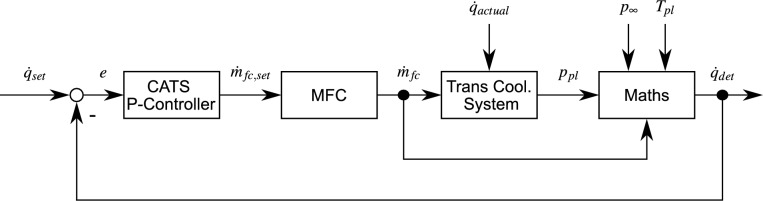
Layout of CATS system.

The resulting control loop is sketched in Fig. 2. The surface heat flux $\dot{q}_{actual}$ acts as the disturbance variable to the transpiration cooling system and causes the plenum pressure to rise from its initial value. The system uses the plenum pressure signal to determine the surface heat flux $\dot{q}_{det}$. By defining $\dot{q}_{set}=0$, the determined surface heat flux equals the control deviation and directly serves as the input into the CATS P-controller. Here, a simple proportional follow-up controller is sufficient. Its output is the set point for the MFC ${\dot{m}}_{fc,set}$, which controls the actual mass flow rate into the transpiration cooling system ${\dot{m}}_{fc}$ accordingly.

In summary, the approach theoretically described in this section allows to automatically adjust the cooling depending on the measured heat flux whereas this heat flux is determined from exclusively non-intrusively acquired values of the system. The prime feature of this controller is that it reacts to the surface heat flux rather than time delayed quantities as e.g. wall temperature. This boosts the protective characteristic of the transpiration cooling technique in the same instance the actual heating affects the surface. Thus, the total heating of the wall is minimized at the lowest possible cost. In theory, the proposed pressure heat flux transformation eliminates a destabilizing positive feedback loop, where the controller output—mass flow rate—significantly influences its input—plenum pressure.

## Experimental transpiration cooling system

**Figure 3:**
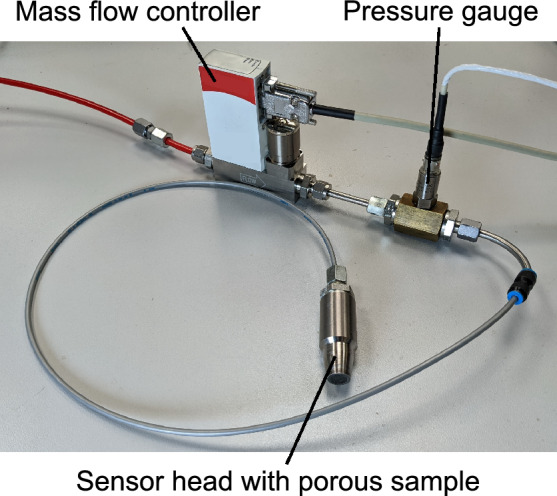
The transpiration cooling system.

The complete transpiration cooling system used in the experiments is shown in Fig. 3. It consists of the sensor head, a pressure gauge, a MFC, and respective tubing between the components. This setup was analyzed and calibrated in a test rig where a known heat flux is applied to the sample using an infrared laser. Furthermore, the controller was tested in a high-enthalpy air flow in the plasma wind tunnel PWK4 of the Institute of Space Systems at the University of Stuttgart. Here, a steady state plasma flow can be established with heat fluxes of several MW/m$^2$^18^.

**Figure 4:**
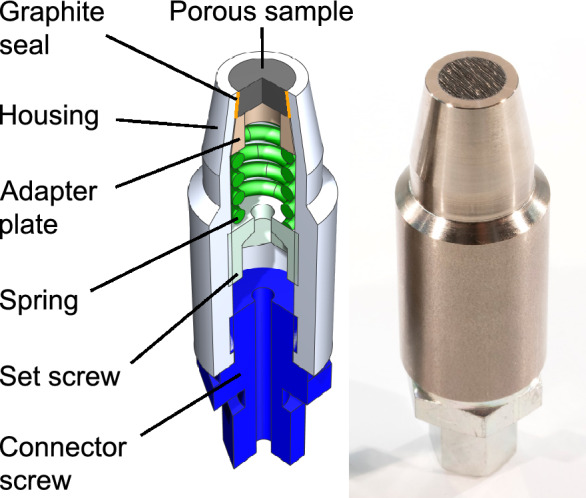
Transpiration cooling sensor head schematic (left) and photo (right).

A schematic and a macro photo of the sensor head are shown in Fig. 4. The porous sample is made of carbon/carbon (C/C). The C/C sample was machined into a truncated conical shape with a draft angle of $10^{\circ }$ and a front diameter of 10.6 mm (smaller diameter of the truncated cone). With a sample length *L* of $7.5\,$mm follows a mean sensor diameter of 11.5 mm and a mean cross sectional area *A* of $1.1\times 10^{-4}$ m$^2$. The Darcy permeability coefficient $K_D$ for the porous sample in this study was determined to be $4\times 10^{-13}\,$m$^2$. The porous sample is pressed into a titanium housing by a set screw. A spring accounts for thermal expansion. A *Sigraflex* graphite foil acts as the sealant between the porous sample and the housing. The sensor head’s total length including the shown connector screw is $81\,$mm and its maximum diameter is $25\,$mm.

A thin tube leads from the connector screw at the back of the sensor head to the pressure gauge and the MFC. The pressure gauge is a *Kulite ETQ-12-375M-5BARA* with an accuracy of $0.025\,$bar. The MFC is a *Bronkhorst FG-201CV-AAD-33-V-AA-000* with a range of $0-42$ mg/s and an accuracy of $0.042\,$mg/s plus 0.5% of the measured value. The analog signals from the pressure gauge and the mass flow controller are recorded using a *Analog Devices AD7682* analog-to-digital converter with an accuracy of $11.7\,$mV. This corresponds to an additional uncertainty in the plenum pressure and flow controller mass flow rate signal of $0.013\,$bar and $0.098\,$mg/s respectively. The MFC is supplied with nitrogen with a constant pressure of $16\,$bar and a gas temperature of $\approx \,295\,$K which is measured using a Pt-100 thermometer. The gas temperature in the plenum typically varies $<1\,$K during a given experiment and is therefore assumed constant. The plenum reaches from the exit of the mass flow controller to the inner surface of the porous sample. The total plenum volume $V_{pl}$ was determined to $15.0\times 10^{-6}\,$m$^3$ for the calibration measurements. The tubing had to be slightly modified for the PWK experiments resulting in a plenum volume of $15.7\times 10^{-6}\,$m$^3$ in the PWK measurement. This small difference was accounted for in the calculation of the mass flow rate through the porous wall with Eq. (9).

## Calibration and verification

With a calibration step, the model parameters of the heat flux determination model given by Eq. (12) are tuned to obtain both an accurate heat flux measurement and a stable controller. The boundary conditions in terms of temporal resolution and controller instability limit are given by the application, in the present case an experiment in a hot gas flow with varying heat flux between 0.2 and 1.4 MW/m$^2$. This calibration requires a well known heat flux at the sensor surface which is provided by a laser diode system.

### Experimental setup for calibration and verification

**Figure 5:**
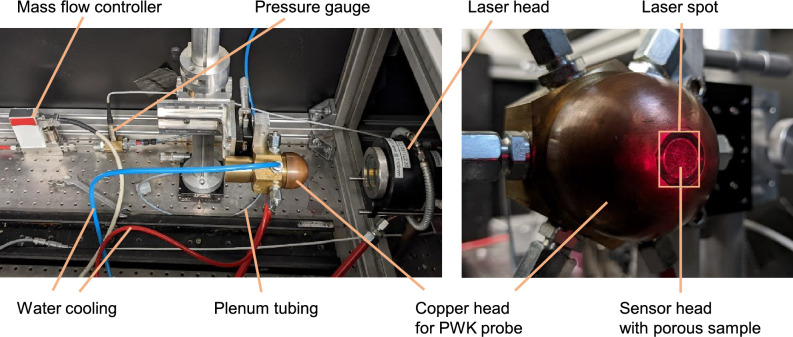
Experimental setup for calibration (left) and visual impression of laser spot on transpiration cooled sensor surface (right).

The experimental setup for calibration and verification is shown in the left photo in Fig. 5. The well-characterized calibration heat flux is provided by a *Laserline LDM 500-100* diode laser with a wavelength of $910\,$nm and a power rise time of $<0.1\,$ms. The relative accuracy of the net heat flux is $5\%$. This includes the uncertainties of laser power of $3\%$, C/C absorption coefficient of $3.5\%$, and laser spot homogeneity and alignment of $1.9\%$. The focusing optics in the laser head expand the laser beam into a square-shaped spot as illustrated in Fig. 5 on the right. To minimize lateral conduction, the radiated area slightly overlaps the porous material’s surface to all sides. If we change the laser power and thus the surface heat flux, the measurement chain is comparable to the wind tunnel experiment which therefore allows us to calibrate the system in this setup.

### Calibration of heat flux determination model

**Figure 6:**
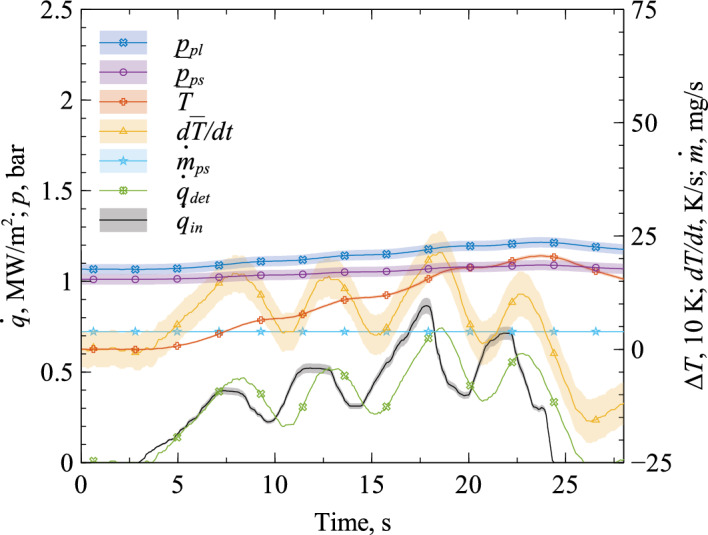
Experimental data for calibration of heat flux determination model: the system’s input is the heat flux $\dot{q}_{in}$, the resulting determined heat flux is ${\dot{q}}_{det}$ which depends on the other plotted variables.

The data of the experiment for the calibration of the heat flux determination model are shown in Fig. 6. In this experiment, we established a constant mass flow rate set point ${\dot{m}}_{fc,set}$ and applied an arbitrary heat flux using the diode laser. The laser’s output power is recorded and calculated into the net surface heat flux $\dot{q}_{in}$. Here, we take the absorption coefficient of C/C $\epsilon =0.85$ into account^3^. We measured the plenum pressure $p_{pl}(t)$, and the actual value of mass flow rate through the MFC ${\dot{m}}_{fc}$, which consequently equals the constant set point, i.e. ${\dot{m}}_{fc} = {\dot{m}}_{fc,set}$. The ambient pressure $p_{\infty } = 0.955\,$bar and the plenum temperature $T_{pl}=295$ K remained fairly constant throughout all calibration experiments. With these measurements, we calculate ${\bar{p}}_{ps}$, ${\dot{m}}_{ps}$, and $\bar{T}$ using Eqs. (8–10) and the look-up table as described in “Theoretical approach”. The temperature’s time derivative $d\bar{T}/dt$ is calculated at each time step $n_t$ using the two-point backward difference $d{\bar{T}}/dt(n_t) = (T(n_t)-T(n_t-1))/\Delta t$ with the time step width $\Delta t$. The time step width was $\Delta t=80\,$ms in all experiments. The plenum pressure’s time derivative is calculated in the same way.

Using the data shown in Fig. 6, the model parameters *a*, *b*, and *c* were fitted to Eq. (12). As a starting point for the fit, we calculated the model parameters using the analytical expressions from Eqs. (11) and the material and geometry data which are summarized in Table 1. Both the parameter fit and the analytical values are given in Table 2.

**Table 1:** Material and geometry data of the porous C/C sample and nitrogen.

Porosity C/C	$\Phi$	12.4%^19^
Effective density C/C	$\rho _{s,eff}$	1375 kg/m$^3$^16^
Heat capacity C/C	$c_{p,s}$	950 J/(kg K)^16^
Heat capacity N$_2$	$c_{p,f}$	1040 J/(kg K)
Specific gas constant for an ideal gas N$_2$	*R*	297 J/(kg K)
Porous sample length	*L*	7.5 mm
Porous sample cross sectional area	*A*	1.11 cm$^2$

**Table 2:** Model parameters for different measurement scenarios.

	*a*	*b*	*c*
Analytical value	$1.0\times 10^4$	$3.3\times 10^{-3}$	$1.2\times 10^6$
Model calibration	$1.6\times 10^4$	22	$4.4\times 10^8$
Closed loop validation	$1.6\times 10^4$	15	$8\times 10^7$
PWK experiment	$1.0\times 10^4$	10	$4\times 10^7$

As can be seen in Fig. 6, the heat flux determination ${\dot{q}}_{det}$ matches the input heat flux ${\dot{q}}_{in}$ fairly well, with a small time delay. The root mean squared error of the ${\dot{q}}_{det}$ curve fit is 0.12 MW/m$^2$. The time delayed reaction of the two signals may be explained by the introduction of moving average filters to the signals of $p_{pl}$, $dp_{pl}/dt$, $d{\bar{T}}/dt$, and $\dot{m}_{fc,set}$. The previous 4, 154, 47, and 41 data points are averaged which amounts to a length of 0.3, 12.3, 3.8, and 3.3 s respectively. Especially the filter for $dp_{pl}/dt$ is quite broad-ranged. These filters were introduced to stabilize the control loop.

**Figure 7:**
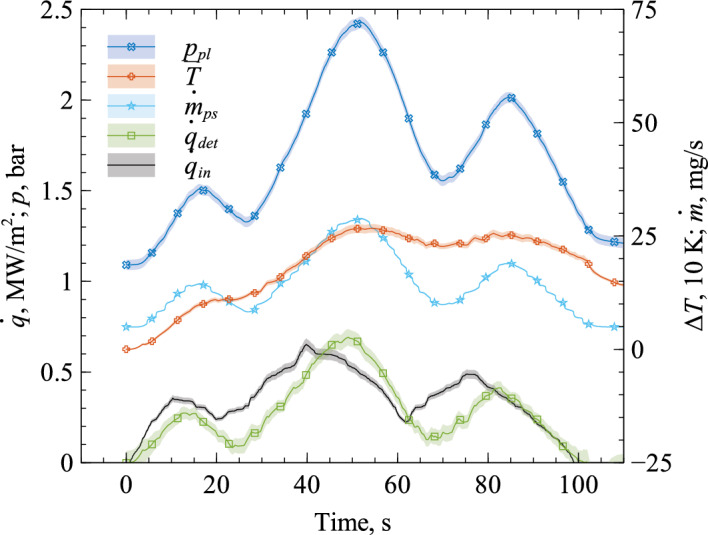
Closed loop verification data with real-time determined heat flux.

**Figure 8:**
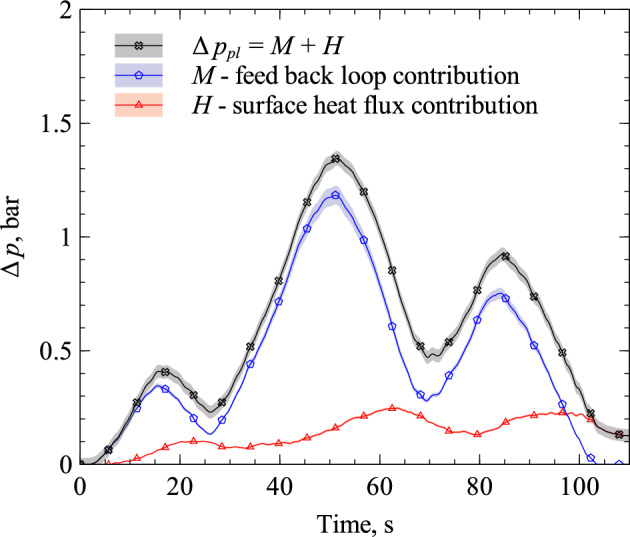
Comparison of the contributions of positive feedback loop *M* and surface heat flux *H* to the input signal $\Delta p_{pl}$.

The uncertainties of the variables were calculated by common methods as described e.g. in^20^. Transparent error bars are given in Figs. 6, 7, 8 and 11. Here, the time derivatives of ${p}_{pl}$ and $\bar{T}$ play a crucial role, because in each of these calculations the uncertainty due to noise is amplified by a factor of $\sqrt{2}/\Delta t\approx 18$. As a result, the measurement noise is amplified significantly, which reflects in large uncertainty for $d\bar{T}/dt$, e.g. up to $\approx 9000\%$ in the calibration experiment. This illustrates the necessity of signal filtering, which we did as described above. The application of filters to a time varying signal has two effects, the favorable reduction of noise and the unfavorable introduction of a bias. In the calculation of the error bars, we take the noise reduction into account by reducing the uncertainty by the factor of $1/\sqrt{N}$ with the filter width *N*. The uncertainty due to the bias cannot be quantified and is not taken into account.

Note that this calibration is conducted at the heat flux levels expected for the experiment, i.e. in the MW/m$^2$ range, which means Eq. (12) is known within the heat flux range of interest. This calibrated model can now be tested using the controller to adjust the mass flow depending on the heat flux level, measured by the pressure gauge.

### Closed loop verification

The aim of the closed loop verification was to verify the found model parameters for the active closed loop CATS controller. This means the set point of the MFC is adjusted by CATS. Since the mass flow rate affects the plenum pressure and the plenum pressure is at the same time the essential input of CATS, the system is a positive feedback loop controller. According to theory, this feedback loop is eliminated by the approach described in “Theoretical approach”, but we observed a slight tendency of the system for instability. The reason for the instability might be a difference in time delay through filtering of the signals for ${\bar{T}}$ and ${\dot{m}}_{ps}$, where the ${\bar{T}}$ signal is subject to broader filters and thus more time delayed. These terms play a role in the last term of Eq. (12), which is the term for energy loss through hot gas motion. For a given heat flux and a rising ${\dot{m}}_{ps}$, the temperature difference ${\bar{T}}- T_{pl}$ decreases. If that decrease is time delayed with respect to ${\dot{m}}_{ps}$, the heat flux is overestimated resulting in a positive feedback loop. The same applies to the second term in Eq. (12). To reduce this effect and achieve a stable controller, we modified the model parameters *b* and *c* slightly. An accurate heat flux determination was maintained.

Figure 7 shows a verification experiment with the closed loop controller, where we used the modified model parameters given in Table 2. As one can see, the system remains stable in this experiment and the heat flux determination ${\dot{q}}_{det}$ matches the input heat flux ${\dot{q}}_{in}$ still sufficiently well. The given error bar of ${\dot{q}}_{det}$ does not contain model uncertainties. The determined heat flux shows a similar time delay as during calibration (cf. Fig. 6). The mass flow rate through the sample increases when heat flux increases, which results in a plenum pressure rise as well. However, it is shown through this verification experiment, that a stable and physical heat flux measurement and control system has been set up. In conclusion, the model parameters (see Table 2) are valid for the present controller settings.

To interpret the positive feedback, i.e. the increase in plenum pressure through mass flow rate increase instead of heat flux increase, the contributions to the pressure signal are analyzed. Therefore, we solve Eq. (10) for $p_{pl}(t)^2-p_{\infty }^2$ and substitute the mass flow rate through the porous sample using Eq. (9). Also, we substitute the mass flow rate through the flow controller, the viscosity, and the mean temperature with $\dot{m}_{fc}(t) = \dot{m}_{fc}(0)+\Delta \dot{m}_{fc}(t)$, $\mu (t)=\mu (0)+\Delta \mu (t)$, and ${\bar{T}}(t)={\bar{T}}(0)+\Delta \bar{T}(t)$ respectively. Here, $\dot{m}_{fc}(0)={\dot{m}}_{ps}(0)$, $\mu (0)$, and $\bar{T}(0)$ are the respective initial values. We yield

14 $$\begin{aligned} p_{pl}(t)^2-p_{\infty }^2{} & {} =\, \frac{2\,L\,R}{K_D\,A}\,\left( \dot{m}_{fc}(0)+\Delta \dot{m}_{fc}(t)-\frac{V_{pl}}{R\,T_{pl}}\frac{dp_{pl}(t)}{dt}\right) \,\Bigl (\mu (0)+\Delta \mu (t)\Bigr )\,\Bigl ({\bar{T}}(0)+\Delta {\bar{T}}(t)\Bigr )\nonumber \\{} & {} =\, \frac{2\,L\,R}{K_D\,A}\,\biggl ({\dot{m}}_{fc}(0)\,\mu (0)\, {\bar{T}}(0) + {\dot{m}}_{fc}(0)\Bigl (\mu (t)\,\Delta \bar{T}(t) + \Delta \mu (t)\,{\bar{T}}(0) \Bigr ) +\left( \Delta {\dot{m}}_{fc}(t)-\frac{V_{pl}}{R\,T_{pl}}\frac{dp_{pl}(t)}{dt}\right) \,\mu (t)\,\bar{T}(t) \biggr ) . \end{aligned}$$

Subtraction of the initial condition

15 $$\begin{aligned}{} & {} p_{pl}(0)^2-p_{\infty }^2=\frac{2\,L\,R}{K_D\,A}\,{\dot{m}}_{fc}(0)\,\mu (0)\, {\bar{T}}(0) \end{aligned}$$

on both sides of Eq. (14) eliminates the ambient pressure and gives

16 $$\begin{aligned}{} & {} p_{pl}(t)^2-p_{pl}(0)^2 = \frac{2\,L\,R}{K_D\,A}\,\biggl ( \left( \Delta {\dot{m}}_{fc}(t)-\frac{V_{pl}}{R\,T_{pl}}\frac{dp_{pl}(t)}{dt}\right) \,\mu (t)\,\bar{T}(t) + {\dot{m}}_{fc}(0)\Bigl (\mu (t)\,\Delta {\bar{T}}(t) + \Delta \mu (t)\,{\bar{T}}(0) \Bigr ) \biggr ) . \end{aligned}$$

We derive an expression of the pressure difference $\Delta p_{pl}(t)$ by substituting

17 $$\begin{aligned} p_{pl}(t)^2-p_{pl}(0)^2 = \bigl (p_{pl}(t)-p_{pl}(0)\bigr )\bigl (p_{pl}(t)+p_{pl}(0)\bigr ) = \Delta p_{pl}(t)\,\bigl (p_{pl}(t)+p_{pl}(0)\bigr ) \end{aligned}$$

into Eq. (16). We get

18 $$\begin{aligned} \Delta p_{pl}(t) = \underbrace{\frac{2\,L\,R}{K_D\,A}\,\cdot \,\frac{\Delta \dot{m}_{fc}(t)\,\mu (t)\, \bar{T}(t)}{p_{pl}(t)+p_{pl}(0)}}_{M} +\underbrace{\frac{2\,L\,R}{K_D\,A}\,\cdot \frac{{\dot{m}}_{fc}(0)\Bigl (\mu (t)\,\Delta {\bar{T}}(t) + \Delta \mu (t)\,\bar{T}(0)\Bigr )-\frac{V_{pl}}{R\,T_{pl}}\frac{dp_{pl}(t)}{dt}\,\mu (t)\,\bar{T}(t)}{p_{pl}(t)+p_{pl}(0)} }_{H} . \end{aligned}$$

On the right side of Eq. (18), the term *M* isolates the effect of the positive feedback loop, because if the mass flow rate is not adjusted by the CATS P-controller then $\Delta \dot{m}_{fc}(t)=0$ and consequently $M=0$. The term *H* is independent of the mass flow rate change. It is driven by the temperature change and is, therefore, a result of the surface heat flux, i.e. the actual signal we intend to utilize for the transpiration cooling control. The individual curves for $\Delta p_{pl}$, *M*, and *H* of the verification measurement data are plotted in Fig. 8 using the formulation from Eq. (18).

It becomes apparent, that the contribution of the positive feedback loop (*M*) to the total plenum pressure difference ($\Delta p_{pl}$) is significantly stronger than that of the surface heat flux (*H*). This is the substantial challenge of a transpiration cooling controller based on the measurement of plenum pressure. This theoretical analysis is confirmed by our experimental research where we found that the usage of the plenum pressure as the control parameter directly results in severe instability. With the proposed approach to transform the plenum pressure into a heat flux information, this difficulty was resolved. CATS is capable to distinguish between the contribution of heat flux and mass flow rate change (*H* and *M* in Fig. 8) and eliminates the instability caused by the positive feedback loop. Thus, we show that this control system is applicable for an automated heat flux reduction through coolant mass flow control based on plenum pressure measurement. With respect to a real flight application, this means that a pressure measurement at the surface and behind a transpiration cooled heat shield are sufficient to automatically control the cooling of that surface.

## Plasma wind tunnel experiment

### Experimental setup of the plasma wind tunnel experiment

**Figure 9:**
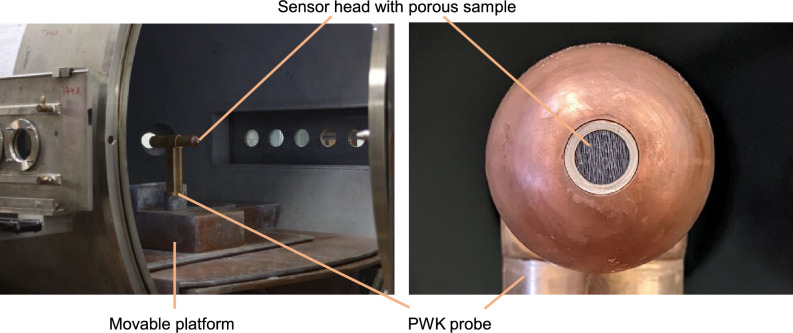
Experimental setup for plasma wind tunnel tests (left) and close up photo of probe head (right).

The final step of this new method is its application in a real heating environment which includes pressure changes and aerothermodynamic effects. The system was tested in the plasma wind tunnel PWK4 at the Institute of Space Systems, University of Stuttgart. Details of the facility are described in Ref.^18^. In this subsection, we describe only the setup for the particular experiment. The experimental setup of the PWK4 test is shown in Fig. 9. The sensor head is mounted into the tip of a probe holder as it is standard for such facilities. The transpiration cooled surface orientation opposes the plasma flow direction. The tube from the back of the sensor head leads through the interior of the PWK probe to the pressure gauge. The pressure gauge and the MFC are located beneath the movable platform under water-cooled copper shields.

**Figure 10:**
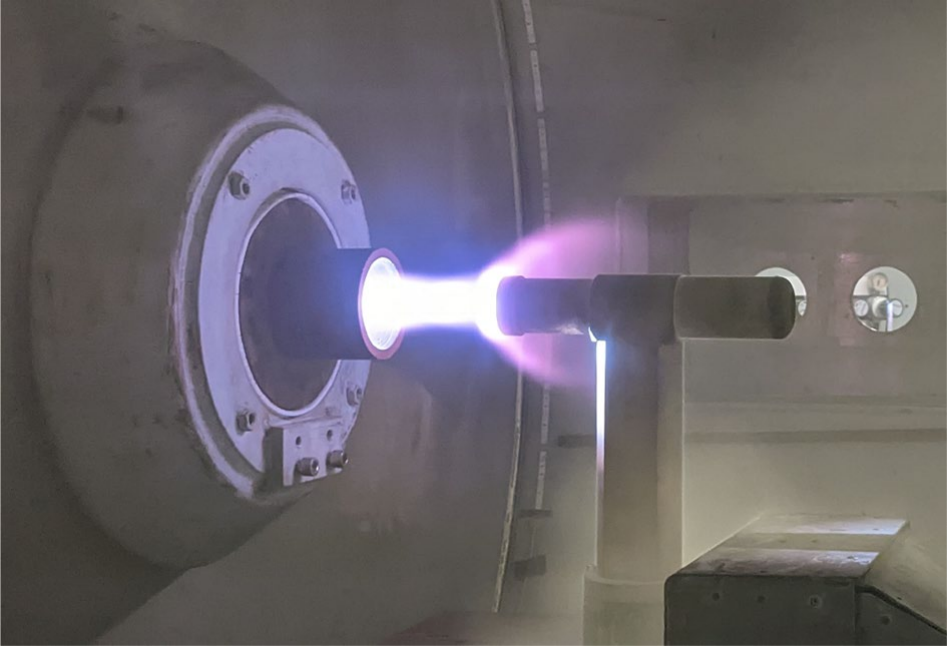
Visual impression of the plasma wind tunnel experiment.

The plasma wind tunnel PWK4 is an arcjet facility^18^. For the present study, we set a flow condition that is regularly used for Earth Reentry Flight investigations, e.g.^18,21,22^. The established condition results in a $\sim 1\,$m long supersonic air plasma jet with an axial variation of heat flux of 0.2–1.4 MW/m$^2$ and total pressure variation of 100–1200 Pa. The lateral profile of the plasma jet is uniform over a diameter of about 100 mm^23^. Thus the whole probe head fits into the uniform region. Figure 10 gives a visual impression of the PWK4 experiments. The facility parameters characterizing this flow condition equal the IRS-Ames-1 condition in^21^.

The ambient pressure that the transpiration cooling system is exposed to changes during the experiment. Initially, the ambient pressure equals the vessel pressure of $p_{tank}=52$ Pa and increases up to roughly 1250 Pa in the plasma flow’s center. This variation is not measured and thus it is not accounted for in the experiments. However, this ambient pressure variation is rather small when compared to the development of plenum pressure, which is $>32{,}000$ Pa. Considering the fact that the pressure variables are squared or cubed in Eqs. (8) and (10), this effect of the ambient pressure variation is considered negligible, which corresponds to an assumption of a constant ambient pressure.

### Results of the plasma wind tunnel experiment

**Figure 11:**
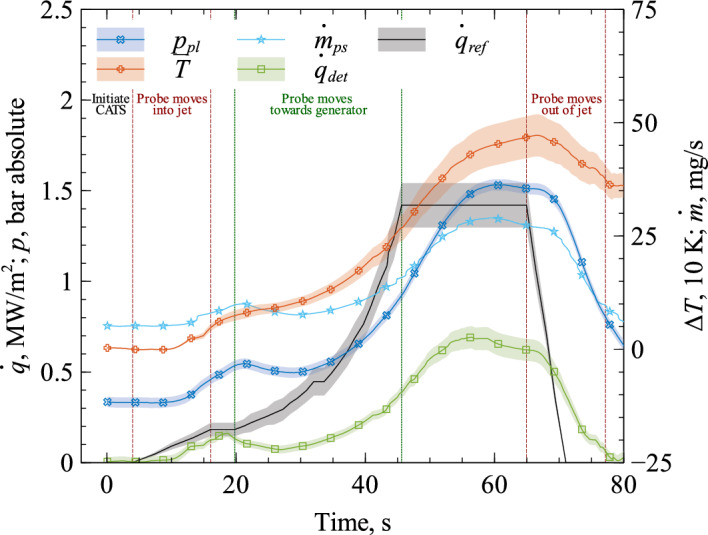
Demonstration of automatic mass flow rate adjustment (CATS) in plasma wind tunnel PWK4 experiment.

The performance of CATS is shown by exposing the front face to a heavily varying heat flux while observing the system’s controller reaction. Figure 11 shows the results of this demonstration. Once the flow condition was set, the system was initiated at $t=0\,$s and ran freely throughout the experiment. The movement of the probe is added in Fig. 11. The closer the probe moves to the generator the higher the heat flux and pressure. During startup, the probe was positioned outside the plasma jet at a distance from the generator nozzle of $490\,$mm. It was first moved quickly in radial direction into the plasma jet center. This means the system was changed from a situation with almost no heating (outside the jet) to high heating (on the centerline) within about 10 s. About 5 s after reaching the centerline, the probe was moved axially toward the plasma generator up to a distance of $90\,$mm. During the axial movement, the probe remained in the plasma flow’s stagnation point. Finally, the probe was moved radially out of the jet.

The black line in Fig. 11 gives a reference heat flux $\dot{q}_{ref}$ at the respective positions in the plasma flow obtained in a separate PWK4 run using a water calorimeter probe with a cold copper surface. For more details on this calorimetric probe, the reader is referred to^21^. Both the reference and the determined heat flux are the net value, i.e. the heat flux that is absorbed by the wall. The reference heat flux shows qualitatively the heat flux level affecting the CATS transpiration cooling sensor head. The reference heat flux in Fig. 11 illustrates that moving radially into the jet leads to a heat flux increase. This is detected by CATS as can be seen from the rising signal of determined heat flux ${\dot{q}}_{det}$ during that phase (cf. Fig. 11). As a consequence, CATS automatically increases cooling, i.e. increases the mass flow rate as seen in ${\dot{m}}_{ps}$. The model parameters were adjusted slightly after the very first experiment to increase stability (see Table 2). Although this adaption introduces a significant error to the real-time heat flux determination, which is like the model uncertainties not contained in the given error bar of ${\dot{q}}_{det}$, the CATS controller functioned nominally as desired. The surface heat flux increases again during the axial movement towards the generator, but with different temporal behavior and over a wider heat flux range (cf. $\dot{q}_{ref}$ in Fig. 11). This is also detected and the CATS controller automatically increases the mass flow rate. Between $46\,$s and $65\,$s, the probe remained in the same position which means a constant flow condition. CATS required about $10\,$s to stabilize to this constant flow condition, which is to be explained by the rather broad-ranging moving average filters introduced in the signal processing of CATS. However, the heat flux signal decreases after this stabilization period, which is an effect of the increased out-blowing. We conclude from this that the demonstration of CATS was successful. Again, throughout this whole experiment, manual intervention was neither required nor foreseen.

## Conclusion

This paper shows a new approach in theory and experiment to automatically adjust the cooling rate of a transpiration cooled wall depending on the actual surface heat flux. The heat flux is measured in-situ from the non-intrusively measured plenum pressure data. The system is meant to boost the protective characteristic of the transpiration cooling in the same instance that the actual heating affects the surface. The proposed model requires the time variant inputs plenum and ambient pressure, mass flow rate through the mass flow controller, and temperature of the inbound coolant temperature. All of these parameters are non-intrusive measurements, thus the porous wall does not require any additional sensor which potentially weakens the performance and/or structural integrity of the heat shield material. We calibrated and verified the model parameters for the determination of surface heat flux using an infrared laser. A cooling adjustment for transpiration systems (CATS) controller was successfully tested in a plasma wind tunnel experiment. This is the first experimental proof of a functional controller for an automated adjustment of the transpiration cooling to the actual surface heat flux.

## Data Availability

The datasets generated during and/or analyzed during the current study are available from the corresponding author on reasonable request.

## References

[1] Kays WM (1972). Heat transfer to the transpired turbulent boundary layer. Int. J. Heat Mass Transf..

[2] Glass DE, Dilley AD, Kelly HN (2001). Numerical analysis of convection/transpiration cooling. J. Spacecr. Rocket..

[3] Boehrk H, Piol O, Kuhn M (2010). Heat balance of a transpiration-cooled heat shield. J. Thermophys. Heat Transfer.

[4] Nield DA, Bejan A (2013). Convection in Porous Media.

[5] Marvin JG, Pope RB (1967). Laminar convective heating and ablation in the mars atmosphere. AIAA J..

[6] Howe, J. T. Hypervelocity atmospheric flight: Real gas flow fields. Tech. Rep. NAS 1.61:1249, NASA Ames Research Center (1990). https://ntrs.nasa.gov/citations/19910011105.

[7] Schneider SP (2010). Hypersonic boundary-layer transition with ablation and blowing. J. Spacecr. Rocket..

[8] Di Martino, G. *et al.* Design of the transpiration cooled fin experiment finex ii on hiflier1. In *HiSST* (Brugge, BE, 2022).

[9] Loehle S, Schweikert S, von Wolfersdorf J (2016). Method for heat flux determination of a transpiration cooled wall from pressure data. J. Thermophys. Heat Transfer.

[10] Hufgard, F. *et al.* Analysis of porous materials for transpiration cooled heat flux sensor development. In *HiSST* (Russia, Moscow, 2018).

[11] Hufgard, F. *et al.* Surface heat flux measurement in transpiration cooled porous materials using plenum pressure data. In *SciTech 2019* (AIAA, 2019).

[12] Hufgard F (2021). Plenum pressure behavior in transiently heat loaded transpiration cooling system. J. Thermophys. Heat Transfer.

[13] Hufgard, F. *et al.* A heat flux sensor based on transpiration cooling. In *HiSST* (Brugge, BE, 2022).

[14] Hufgard, F., Loehle, S. & Fasoulas, S. Heat flux determination from pressure data in transpiration cooling experiment. In *ICIPE* (2022).

[15] Hufgard, F., Duernhofer, C., Fasoulas, S. & Loehle, S. A transpiration cooled heat flux sensor utilizing plenum pressure: Measurement in high enthalpy flow. In *FAR—2nd International Conference on Flight Vehicles, Aerothermodynamics and Re-entry Missions and Engineering* (ESA Publications Division, 2022).

[16] Schweikert, S. Ein Beitrag Zur Beschreibung Der Transpirationskühlung an Keramischen Verbundwerkstoffen. Ph.D. thesis, Universität Stuttgart, Institut für Thermodynamik der Luft- und Raumfahrt (2019).

[17] Scheidegger AE (1963). The Physics of Flow Through Porous Media. Heritage.

[18] Loehle S (2021). Assessment of high enthalpy flow conditions for re-entry aerothermodynamics in the plasma wind tunnel facilities at IRS. CEAS Sp. J..

[19] Selzer, M., Schweikert, S., Boehrk, H., Hald, H. & von Wolfersdorf, J. Comprehensive c/c sample characterizations for transpiration cooling applications. Tech. Rep., Transregio40-Annual report 2016, pp. 61–72 (2016).

[20] Taylor JR (1997). An Introduction to Error Analysis: The Study of Uncertainties in Physical measurements.

[21] Loehle, S., Nawaz, A., Herdrich, G. & Fasoulas, S. Comparison of heat flux gages for high enthalpy flows—Nasa Ames and IRS. In *46th Aerodynamic Measurement Technology and Ground Testing Conference* (AIAA, 2016).

[22] Leiser, D. *et al.* Spacecraft material tests under aerothermal and mechanical reentry loads. In *SciTech 2019* (AIAA, 2019).

[23] Loehle, S. Untersuchung von Wiedereintrittsplasmen Mit Hilfe Laserinduzierter Fluoreszenzmessungen. Ph.D. thesis, Universität Stuttgart, Institut für Raumfahrsysteme (2006).

